# Advances in targeting cancer-associated fibroblasts through single-cell spatial transcriptomic sequencing

**DOI:** 10.1186/s40364-024-00622-9

**Published:** 2024-07-29

**Authors:** Pin Lyu, Xiaoming Gu, Fuqi Wang, Haifeng Sun, Quanbo Zhou, Shuaixi Yang, Weitang Yuan

**Affiliations:** Department of Colorectal Surgery, The First Affiliated Hospital of Zhengzhou University, No. 1 Jianshe East Road, Erqi District, Zhengzhou, 450000 Henan China

**Keywords:** Single-cell spatial transcriptomic sequencing, Cancer associated fibroblast, Drug resistance, Angiogenesis and metastasis, Metabolic reprogramming

## Abstract

Cancer-associated fibroblasts (CAFs) are the major components of the tumor microenvironment and are related to tumor proliferation, metastasis, relapse, and drug resistance. With the development of sequencing technologies, single-cell RNA sequencing has become a popular method for identifying CAFs in the tumor microenvironment. Whereas the drawbacks of CAFs, such as the lack of a spatial landscape, still exist, recent research has utilized spatial transcriptomics combined with single-cell RNA sequencing to address this issue. These multiomics analyses can resolve the single-cell resolution problem in spatial transcriptomics. In this review, we summarized the recent literature regarding the targeting of CAFs to address drug resistance, angiogenesis, metabolic reprogramming and metastasis in tumor tissue.

**Supplementary Information:**

The online version contains supplementary material available at 10.1186/s40364-024-00622-9.

## Background

With respect to tumor initiation, a considerable number of studies is associated with the “seed and soil” hypothesis proposed by Stephen Paget over a century ago [1]. The last two decades have seen a growing trend toward the tumor microenvironment, which is one of the most frequently noted factors in tumor development, proliferation, metastasis, and relapse.

There are many components of the tumor microenvironment, such as tumor infiltrating lymphocytes (TILs), natural killer cells, tumor infiltrating dendritic cells (TIDCs), tumor-associated macrophages (TAMs), tumor-associated neutrophils (TANs), cancer-associated fibroblasts (CAFs), myeloid-derived suppressor cells (MDSCs), distinct proangiogenic factors and immune checkpoint biomarkers [2]. Traditionally, CAFs have been thought to interact with the cancer cells, having tumor-promoting effects; however, accumulating evidence indicates that CAFs have the apparent tumor-suppressive functions [3, 4]. Moreover, among the whole subgroup of the tumor environment, CAFs account for almost 70% [5], and they can also participate in extracellular matrix remodeling, chemoresistance; radio-resistance; immune evasion and modulation; angiogenesis; and metabolism by secreting various of chemokines, exosomes, cytokines and other effector molecules [6–8].

**Fig. 1:**
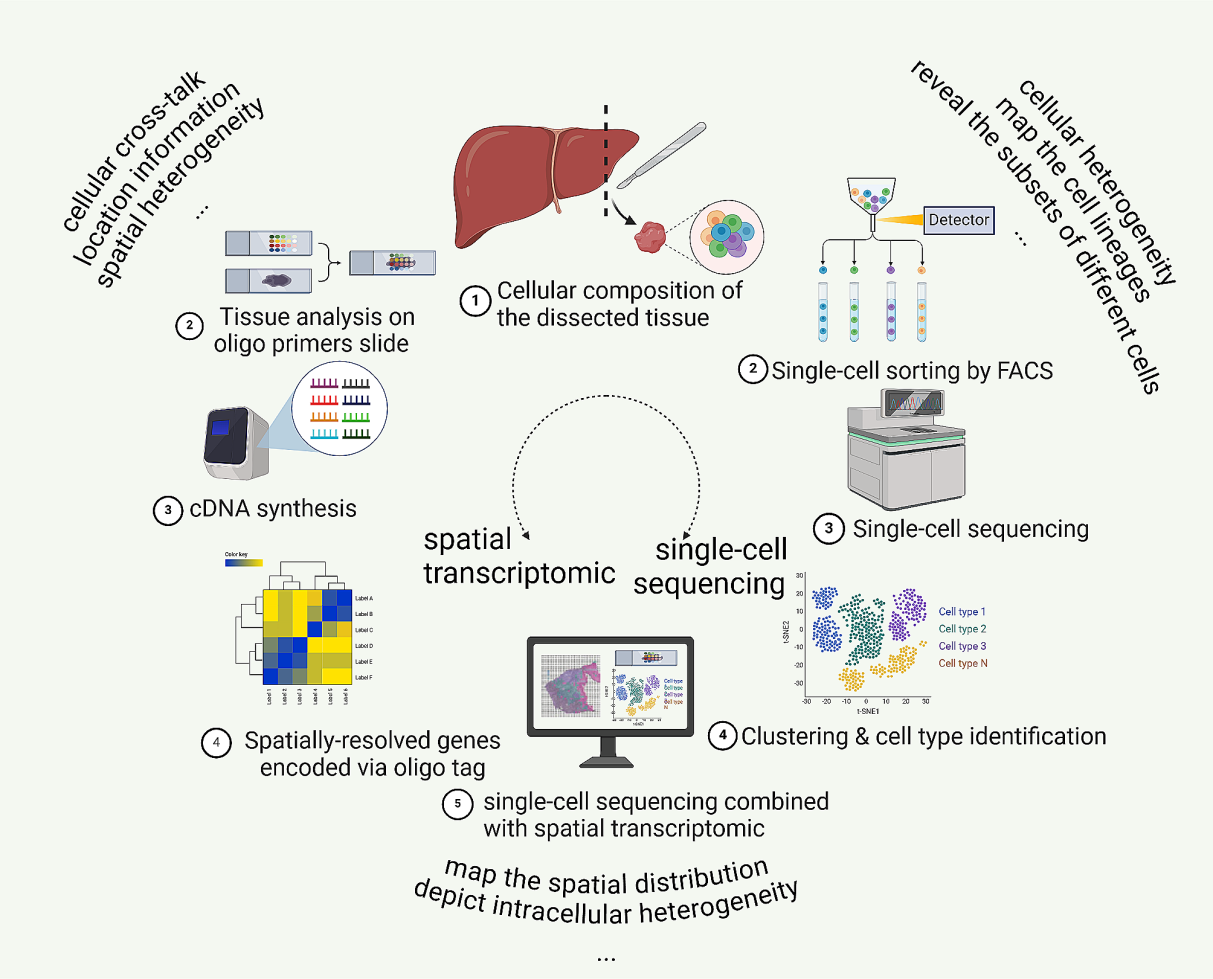
single cell RNA sequencing combined with spatial transcriptomics. Single cell sequencing could research tumor heterogeneity at cellular level, and spatial transcriptomics revels spatial information in tumor tissue. Single-cell spatial transcriptomic sequencing combined the merits of both. (created with biorender.com.)

Increased knowledge of their proportion, heterogeneity, and origin, among other information, at the individual cell level would contribute to a deeper understanding of CAFs. Like separating individual components of a salad, single-cell RNA sequencing (scRNA-seq) can dissociate whole tissues into individual cells [9, 10]. However, this technology does not provide spatial information or address cross-talk in the TME. Therefore, after the wave of single-cell analysis, spatial transcriptomics (ST) has become an important method in the last decade [9, 11]. In addition to overcoming the limitations of scRNA-seq, ST is better for mapping and exploring different functional regions and intercellular interactions in the TME at the two-dimensional level [12, 13]. With increasing throughput and scale, ST produces large amounts of RNA imaging data, and a newly developed computational framework, Bento, was published for the subcellular analysis of ST data [14]. Recently, whole-transcriptomic digital spatial profiling (DSP) revealed three multicellular colonies in PDAC, i.e., treatment enriched, squamoid/basaloid and classical colonies, providing promising therapeutic targets [15]. For colorectal cancer, combined with the ssGSEA algorithm, ST can be used to determine cell types such as dendritic cells, NK cells, monocytes, and epithelial cells in different regions [16]. In addition, according to an ST analysis, high expression of MMP14 has been shown to be associated with tumor progression, poor prognosis, and CAFs and TAMs in tumor tissue [17]. Moreover, ST results showed that CAFs induce glycoprotein nonmetastatic B (GPNMB), which promotes the invasion and migration of tumors in breast cancer [18]. Overall, the combined application of ST and scRNA-seq to explore CAFs allows not only for spatial distribution mapping but also for an assessment of cellular heterogeneity [19, 20]. (Fig. 1)

**Fig. 2:**
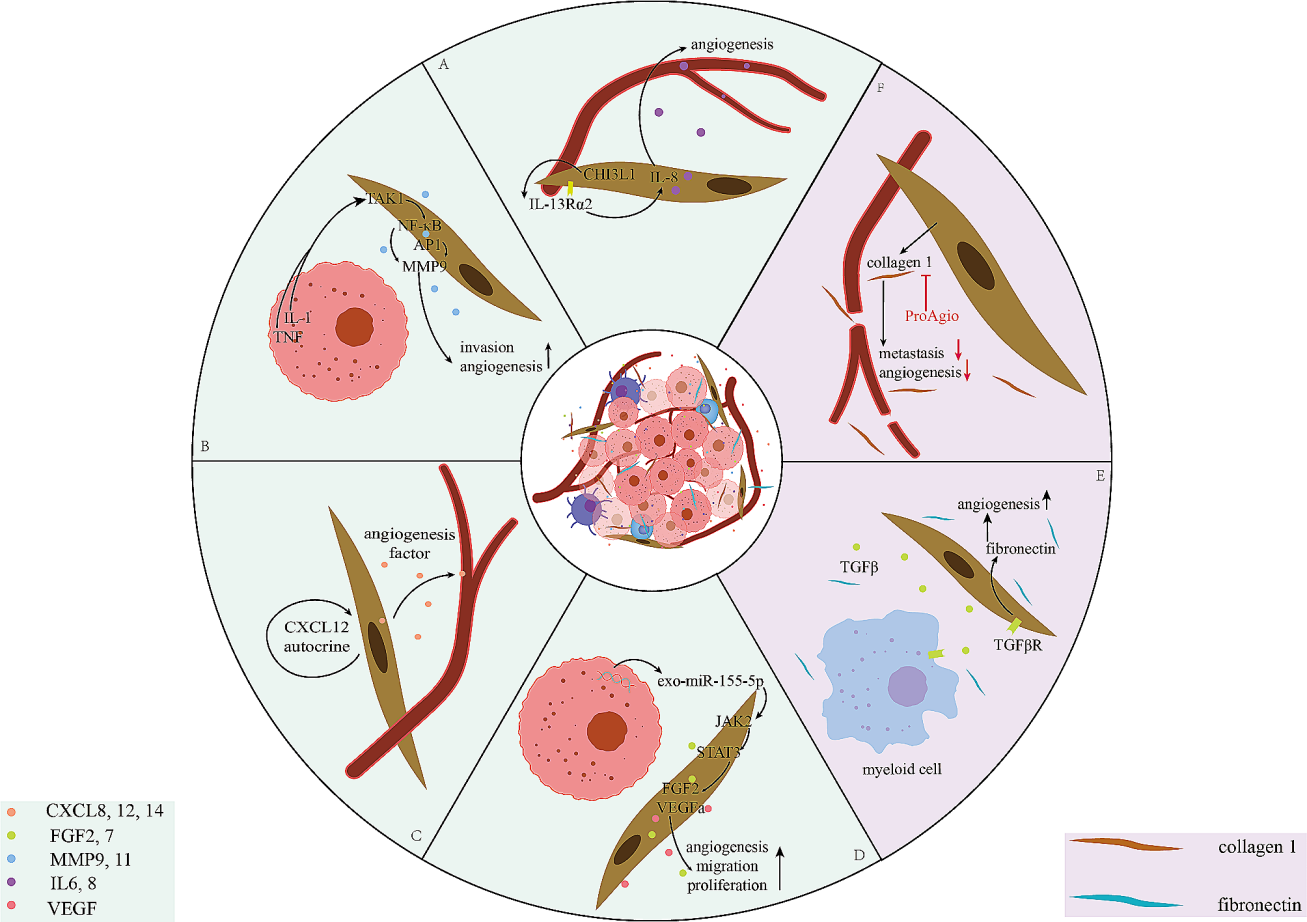
CAFs with angiogenesis through cytokine factors and ECM. **A** Upregulated by CHI3L1/IL-13Rα2 axes, CAFs promotes angiogenesis in IL-8. **B** Activated by IL-1/TNF, CAFs could stimulate TAK1-NFκΒ/AP1-MMP9 signaling which promotes tumor angiogenesis and invasion. **C** In some case, it is important for CAFs to secret angiogenesis factor via the autocrine of CXCL12. **D** In melanoma, miR-155-5p induces tumor angiogenesis and migration in CAFs through JAK2/STAT3 signaling pathway. **E** During ECM formation, myeloid cell upregulates TGFβ promoting angiogenesis in CAFs. **F** Reduced by ProAgio, CAFs decreases tumor angiogenesis and promotes apoptosis

Although extensive research involving single-cell analysis has been carried out for the tumor environment, a complete picture with the comprehensive cellular information is lacking. Owing to the proteomic and epigenomic heterogeneity of the tumor environment, comprehensive information obtained through single-cell multiomics is important [21]. Recently, Cle´mence Henon et al. revealed that the EWSR1::WT1 transcription factor is associated with the prognosis of desmoplastic small round cell tumor patients and customized therapies via single-cell multiomics analysis [22]. Technologies based on genetics, transcriptomics and proteomics have expanded rapidly in recent years, and TME targets and tumor cellular biomarkers can be identified directly by advanced clinical therapy [23]. Through scRNA-seq, spatial transcriptomics, proteomics, Junpeng Fan et al. identified that in cervical squamous cell carcinoma, the MP6/7 score was associated with the immunotherapy response [24]. In addition, metabolic pathway related targets and metabolic enzymes can be utilized in advanced therapeutic strategies, while spatially resolved metabolomics methods can be used to identify genes in their native state [25, 26]. Whereas, single-cell multiomics studies have revealed different challenges, mainly in terms of insufficient sample sizes, a lack of validation in different cancer types [22, 24, 26, 27]. Overall, studies have indicated that multiomics analysis is more beneficial for obtaining a comprehensive understanding of cellular process than any single omics analysis is [28, 29].

The TME, as described above, is a key factors in drug resistance, immunotherapy targeting and tumor progression and metastasis. To date, a considerable number of single-cell multiomics studies of the TME have been published. Furthermore, many studies regarding CAFs, which are a hot topic of TME and cancer research, have been conducted [30].

## CAFs with angiogenesis

As the hallmark of malignant tumors in solid tissue, many cell types play the significant roles in angiogenesis, such as lymphocytes, NK cells, pericytes, CAFs and TAMs [31]. CAFs secrete proangiogenic factors directly and produce ECM indirectly to regulate angiogenesis.

### Proangiogenic factors

**Fig. 3:**
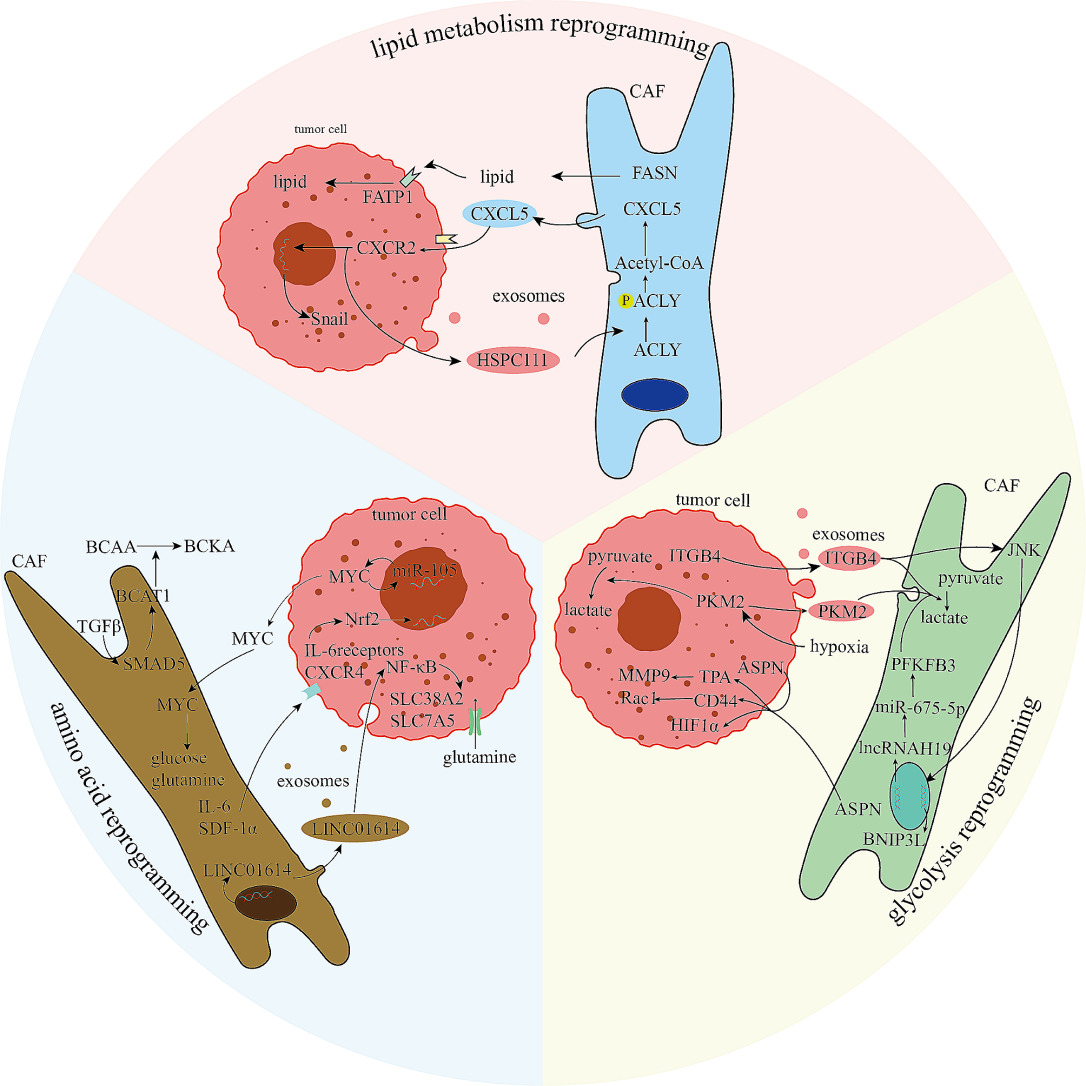
CAFs with glycolysis reprogramming, amino acid reprogramming and lipid metabolism reprogramming. In **glycolysis reprogramming**, we have described five related signaling pathways between CAFs and tumor cells. ASPN, secreted from tumor cell and CAFs, induces glucose metabolism through HIF1α and invasion through CD44/Rac1 and TPA/MMP9 axis. Under the hypoxia, both PKM2 and ITGB4 promote Warburg effect in tumor cell and CAFs in different pathways. Besides, through BNIP3L gene and lncRNA H19, JUN increases CAFs lactate in breast cancer, as well as PFKFB3 promotes Warburg effect in oral cancer. In **amino acid reprogramming**, four signaling pathways have been summarized. In lung cancer, LINC01614 could promote SLC7A5 and SLC38A2 expression for glutamine in cancer cells. And in pancreatic cancer, secreting IL-6 and SDF-1α, CAFs regulates glutamine metabolism in tumor cell via Nrf2. Another signaling pathway indicates TGF-β/SMAD5/BCAT1 axis to regulate branched chain amino acids. In addition, miR-105 and MYC in CAFs activate each other for metabolic reprogramming. In **lipid metabolism reprogramming**, FASN, secreted from CAFs, reprograms lipid metabolism in CRC tumor cell. And HSPC111 also promotes lipid reprogramming in CRLM through CXCL5/CXCR2/ACLY signaling pathway

Secreted from CAFs, different kinds of cytokines, such as the CXCL family, FGF, WNT2, VEGF, and HGF, promote angiogenesis in solid tumors. Recently, studies have demonstrated that CXCL8(IL-8) is a proangiogenic chemokine in pancreatic cancer and TNBC, playing a role through HUVECs [32, 33]. In addition, CXCL12 is another important angiogenesis-promoting factor in lung cancer, prostate cancer and melanoma. CXCL12 expression is higher in healthy tissue (C-MSCs) than tumor tissue (T-MSCs) [34]. In FAP-positive tumor stromal cells, CXCL12 induced angiogenesis via the CXCL12-CXCR4 axis in melanoma and prostate cancer, findings that were authenticated separately [35, 36]. (Fig. 3C) Moreover, in prostate cancer, NIH-CXCL14 has been shown to stimulate angiogenesis [37].

As one of the proangiogenic factors in CAFs, FGF2 is a promising target for normalizing vessel tubes in lung cancer, breast cancer, melanoma and pituitary tumors [38–41]. In addition, some studies have indicated that FGF2 regulates angiogenesis through the JAK2/STAT3 signaling pathways in both lung cancer and melanoma [38, 42]. (Fig. 3D) In addition, another study demonstrated that FGF7 promoted angiogenesis in breast cancer cells and HUVECs via crosstalk between CAFs and tumor cells [41].

Like tumor proliferation and invasion, angiogenesis can be induced by ECM stiffness and degradation, and MMP-related pathways play significant roles in the degradation of the ECM around ECs to promote neovessel formation [43]. In addition, MMP11 expression is increased through different pathways in T-MSCs in lung cancer as well as in breast cancer via coculture with mononuclear inflammatory cells [34, 41]. In addition, high expression of MMP9 has been detected in breast cancer, having similar effects as those observed in oral squamous cell carcinoma, and regulates the stimulation of vascularization via the MAPK-AP1 and TAK1-RELA axes [44, 45]. (Fig. 3B)

Secreted from CAFs, both Il-6 and Il-8 are proangiogenic factors, and Il-6 induces proangiogenic effects via the IL-6/STAT3/NF‐κB positive feedback loop in breast cancer and is regulated by WNT2 in CRC [41, 46, 47]. In addition, studies have demonstrated that the Il-8(CXCL8)/CXCR2 axis is involved in tumorigenesis by promoting angiogenesis in metastasized pancreatic cancer and TNBC [32, 33, 48] (Fig. 3A).

As demonstrated by a considerable number of recent studies, vascular endothelial growth factor (VEGF) supports angiogenesis to promote tumor growth [39, 41, 49]. Herein, we summarized the related articles that address downstream VEGF effects, upstream VEGF effects and anti-VEGF effects. VEGF inhibits the expression of apoptotic cytokines and induces enzymes involved in ECM degradation [50]. However, exo-miRNAs promote proangiogenic factor in melanoma and lung cancer via miR-155-5p and by miR-210 via the JAK2/STAT3 signaling pathway, respectively [42, 51]. (Fig. 3D) In colon cancer, VEGFA expression is modulated by CHI3L1, similar to IL-8 secretion, and is altered by p53 status [52, 53]; however, its secretion can be reduced by eicosapentaenoic acid (EPA) during angiogenesis, similar to the ability of EPA to inhibit the secretion of IL-6 [54]. Research on anti-VEGF factors has focused primarily on bevacizumab, which acts against VEGF by neutralizing sEVs [55, 56].

### Extracellular matrix (ECM)

Unlike proangiogenic factors, component proteins, such as proteoglycans, periostin, tenascin, fibronectin and collagen, have been shown to increase ECM stiffness and remodel the ECM [57]. Studies have demonstrated that both ATF4 and ProAgio are correlated with angiogenesis in melanoma, lung cancer and breast cancer via the expression of collagen I [58, 59]. (Fig. 3F) In addition, we also reported that tumor angiogenesis involves the TGFβ-fibronectin axis via ECM formation and tumor-fibroblast crosstalk in breast cancer [60, 61] (Fig. 3E).

## CAFs with metabolic reprogramming

As the process of changing one cell fate to another, reprogramming affects in tumor progression, metastasis, proliferation and invasion in different cancers. In this review, we summarize advanced research in the following categories: glycolysis reprogramming, amino acid metabolism reprogramming and lipid metabolism reprogramming.

### Glycolytic reprogramming

Aerobic glycolysis, also known as the Warburg effect, plays a major role in tumor glucose metabolism in digestive cancer, breast cancer, and lung cancer. Some studies have focused on the reverse Warburg effect in CAFs, and others have focused on lactate utilization in cancer cells [62–65].

Recently, studies have demonstrated that glycolytic reprogramming in CAFs is involved in tumor progression and heterogeneity in breast cancer via different modes, such as cancer cell-secreted exo-miR-105 promoting MYC expression, cancer cells overexpressing ITGB-4; and normal fibroblasts overexpressing HIF-1α [66–68]. In lung cancer, hypoxia-induced exosomal PKM2 regulates the Warburg effect in CAFs, and ROS, TGF-β and GFPT2 reprogram metabolism by increasing aerobic glycolysis [69–71]. In addition, we found that the MAPK and ERK1/2 signaling pathways are involved in glycolysis reprogramming in CAFs of oral cancer through H10/miR-675-5p/PFKFB3 and CAV-MCT4/MCT1 respectively [72, 73]. In ovarian cancer, LPA secreted from cancer cell induces aerobic glycolysis reprogramming via NOX1, ROS and HIF1α [74, 75].

**Fig. 4:**
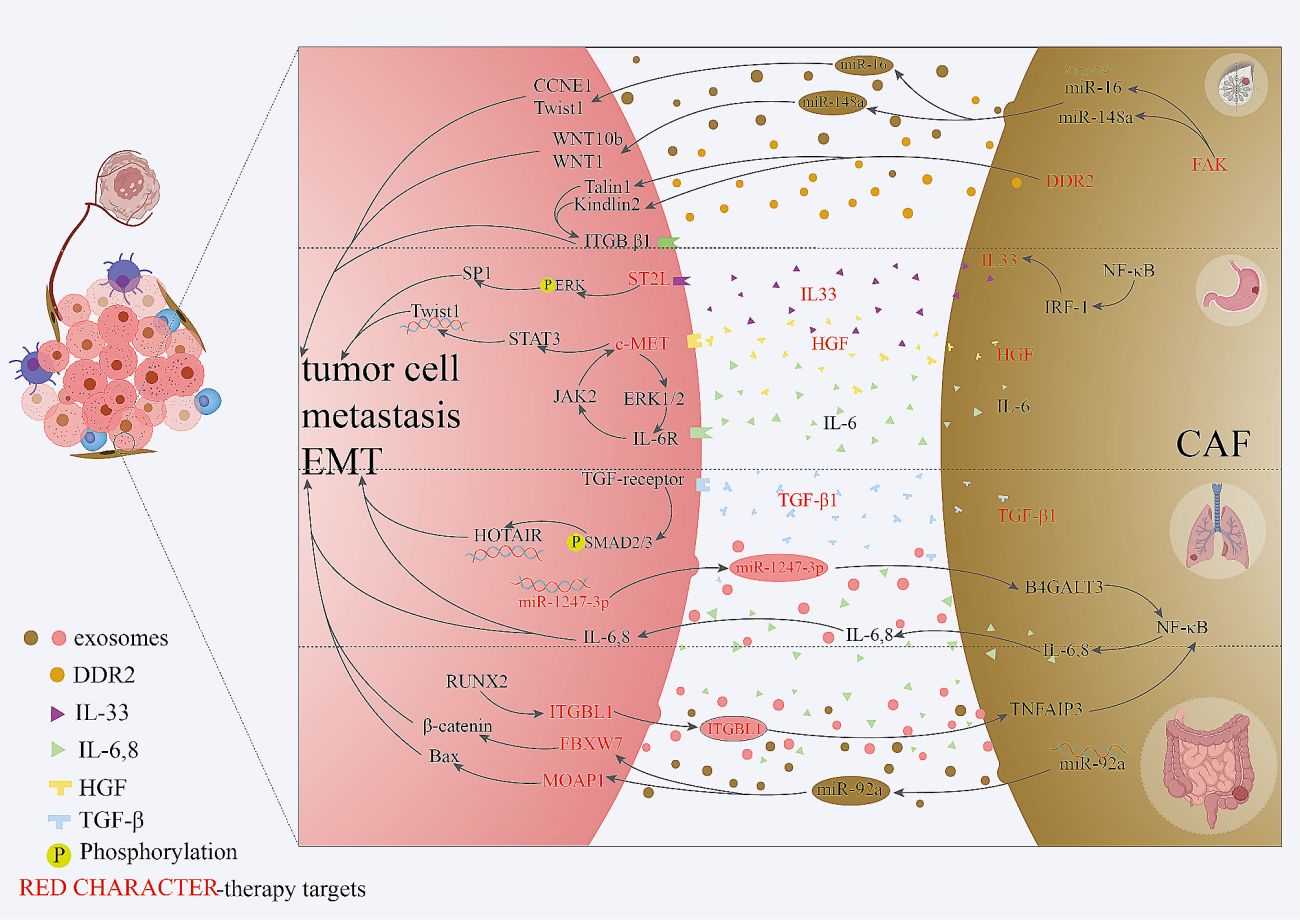
CAFs with metastasis in lung cancer, gastric cancer, colorectal cancer and breast cancer. In **gastric cancer**, CAFs promotes tumor cell metastasis through IL33/ST2L/SP1 and HGF/c-MET/STAT3 signaling pathways. In **breast cancer**, DDR2 and FAK are two therapy targets in tumor metastasis through miR-16/miR-148a and ITGB β1. In **lung cancer**, TGF-β1 promotes metastasis via SMAD2/3-HOTAIR axis and NFκΒ, which activated by miR-1247-3p, increase metastasis through IL-6/8. In **colorectal cancer**, secreted from CAFs, miR-92a increase tumor metastasis and drug resistance through FBXW7 and MOAP1. CAFs can also promotes metastasis via IL-6/8, which activated via RUNX2/ITGBL1/ NFκΒ axis. (created with biorender.com.)

In gastric cancer, the LDHA and ENO2 genes as well as ASPN, are related to the reprogramming of the Warburg effect and anaerobic glycolysis, respectively, in cancer cells [76]. In addition, normal fibroblasts reprogram glucose metabolism into aerobic glycolysis and secrete ROS and MCT4 in oral squamous cell carcinoma [77] (Fig. 4).

### Amino acid metabolism reprogramming

As major metabolites, amino acids are essential for tumorigenesis and metastasis. Moreover, as a major amino acid, glutamine is essential for metabolic reprogramming [62, 64, 78]. Herein, we summarize recent studies of the reprogramming of stromal cells and cancer cells. In CAF-derived exosomes, LINC01614 inhibited LUAD growth and reprogrammed glutamine addiction in tumor cells by activating NF-κB to upregulate the expression of the glutamine transporters SLC38A2 and SLC7A5 [79]. In addition, other studies have indicated that CAFs regulate glutaminolysis in CRC and prostate cancer via distinct pathways, such as glutamine synthetase in CRC and Ras, which activates micropinocytosis, in prostate cancer [80, 81]. Additionally, in pancreatic cancer, stromal cells have been shown to regulate glutamine metabolic reprogramming in PDAC cells through the TGF-β/SMAD5 axis, which targets BCAT1, SDF-1a and IL-6 secretion activated by Nrf-2 respectively [82, 83].

However, resistance to glutamine deprivation in stromal cells has been demonstrated by p62-mediated polyubiquitination via upregulated ATF4 expression [84]. In addition, the glutamine metabolism has been shown to be reprogrammed by CAFs through the secretion of miR-105 from cancer cells, promoting tumor growth and metastasis in many solid cancers [68, 85] (Fig. 4).

### Lipid metabolism reprogramming

Previous studies have rarely investigated the lipid metabolism of CAFs; however, recent studies have focused on the principal role of lipid metabolism reprogramming in tumorigenesis, metastasis and potential therapeutic targets [62, 64, 86].

With respect to lipid metabolism, most studies have focused on how lipid metabolic reprogramming affects CAFs. HSP111 reprograms the metabolism of CAFs and promotes CRLM by phosphorylating ACLY to increase H3K27 acetylation and the expression of components of the CXCL5-CXCR2 axis [87]. Additionally, in CRC, iCAFs are correlated with lipid metabolism, and FASN in CAFs promotes tumor migration by lipid metabolism reprogramming [16, 88]. In prostate cancer, the reprogramming of lipid metabolism and amplification of MTOCs have been shown to increase CAF plasticity [89].

However, few studies have investigated lipid metabolism in stromal cells and tumor cells. TNBC-derived CAFs can reprogram monocytes into lipid-associated macrophage (LAMs) via the CXCL12-CXCR4 axis, suppressing immune response such as T cell activation [90] (Fig. 4).

## CAFs with metastasis

CAFs are the major stromal cells in tumor tissue, and their crosstalk with TAMs, TANs, epithelial cells and cancer cells regulates tumorigenesis, invasion, drug resistance and metastasis [91]. Next, we summarize studies of the association between CAFs and metastasis in different cancer types.

### Gastric cancer

Secreting numerous cytokines in the TME, CAFs remodel the tumor stroma in gastric cancer, supporting tumor progression, angiogenesis and metastasis. Herein, we summarize recent studies on tumor cell metastasis and lymph node metastasis.

TNC and twist1 are regulators of many malignant tumors, such as ESCC and CRC, and a recent study revealed that their expression was related to lymph node metastasis in gastric cancer patients [92, 93]. Yang et al. also demonstrated that the expression of KLF5 was associated with lymph node metastasis via activation of the CCL5/CCR5 axis [92]. In addition, other studies have indicated that lymph node metastasis in gastric cancer is associated with the expression of CD9 and the downregulation of FGF9 expression in CAFs [94, 95].

**Fig. 5:**
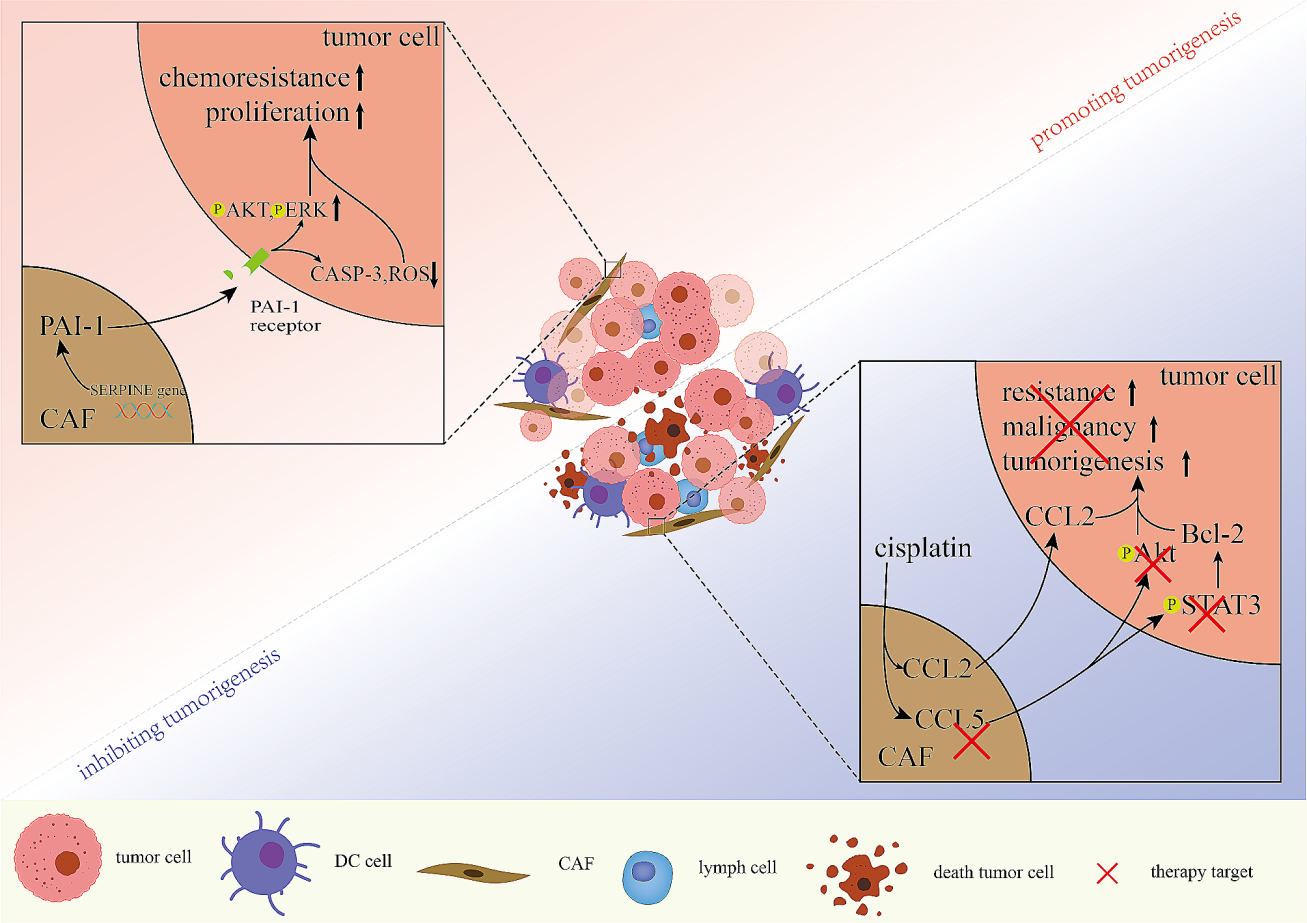
CAFs with drug resistance in promoting tumorigenesis and inhibiting tumorigenesis. Secreting PAI-1, CAFs promoting tumor proliferation and chemoresistance through increasing AKT/ERK and decreasing CASP-3/ROS. And targeting CCL5-Akt/STAT3 axis could reduces drug resistance and tumorigenesis

Recently, CAFs were shown to promote the metastasis of gastric cancer via the TNF-α/IL-33/ST2L signaling pathway and the CAF-derived HGF and IL-11/MUC1 signaling pathways [96–98]. However, another study indicated that exo-miR-139 derived from CAFs inhibited the tumor progression and metastasis of gastric cancer by decreasing MMP11 expression, indicating that CAFs have bidirectional functions [99] (Fig. 5).

### Colorectal cancer

Colorectal cancer (CRC) is the world’s most lethal cancer worldwide, and colorectal metastasis (CRLM) is one of the awkward problems in CRC therapy.

A disintegrin and metalloproteinase (ADAM) and endoglin are two different factors expressed in CAFs that promote CRLM via ADAM10 levels in serum and endoglin-BMP9 axis signaling [100, 101]. In addition, the crosstalk between cancer cells and CAFs via TRAIL-BMP2 has been shown to maintain a feedback loop in a CRLM mouse model [102]. This crosstalk could lead to the development of promising treatments or serve as a predictor of CRC metastasis. The expression of exo-miR-92a-3p is related to CRLM through regulating downstream FBXW7 and MOAP1 expression in CAFs [103].

However, Ruixiao Li et al. have demonstrated that Jianpi Jiedu Recipe (JPJDR) could reduce CRLM through ITGBL1/TNFAIP3/ NF-κB signaling in CAFs [104] (Fig. 5).

### Lung cancer

Lung metastasis is one of the main causes of lung cancer-related deaths worldwide, and lung cancer metastasis is also a key problem in tumor therapy and tumor relapse.

Regulated by miR-1247-3p, CAFs have been shown to induce CCL5 expression through the activation of the HIF1α/ZEB1 axis and the β1 integrin/NF-κB axis to promote lung metastasis in HCC [105, 106]. In addition, researchers have demonstrated that the TGF-β1/HOTAIR signaling pathway and the integrin α2β1/TGF-β axis were promote lung metastasis in patients with breast cancer and salivary adenoid cystic carcinoma, respectively [107, 108].

Other studies have demonstrated that several biomarkers and cytokines derived from CAFs, such as vimentin, FAPα, and HMGB1, are positively related to lung tumor metastasis [109–111]. However, Bin Xue et al. demonstrated that miR-200 expression is associated with lung cancer prognosis and metastasis via the induction of Notch activation in CAFs [112] (Fig. 5).

### Breast cancer

As one of the most common malignant cancers in women, breast cancer is more common than lung cancer, colorectal cancer and thyroid carcinoma. Herein, we have summarize studies that address the promotion and inhibition of metastasis signaling in breast cancer.

Both the NLRP3/IL-1β axis and DDR2 have been shown to contribute to the cancer metastasis in CAFs in vivo and are promising therapeutic targets [113, 114]. In addition, exosomal miRNAs derived from CAFs, such as miR-500a-5p, miR-18b and miR-222, promote metastasis whereas miR-16 and miR-148a expression suppresses metastasis [115–118]. Other studies have revealed that the TGF-β1/HOTAIR and IL-3/integrin β3-p38 MAPK axes promote tumor metastasis [108, 119].

However, CAFs have bidirectional characteristics, i.e., promote tumorigenesis and inhibit tumor metastasis. Research has shown that p85α expression suppresses tumor metastasis via Wnt10b signaling, and that CCM3 secretion reduces metastasis through YAP/TAZ signaling [120, 121] (Fig. 5).

### CAFs with drug resistance

Increasing evidence has shown that CAFs play a key role in drug resistance through signal transduction pathways, drug delivery and acceptance systems, and DNA damage repair. Drug resistance induced by CAFs can be found in many kinds of solid tumors, such as breast cancer, pancreatic cancer, and lung cancer. However, we will address how CAFs promote tumorigenesis through TGF, IL-6, CCL, PAI, NRG1 and some exosomes and inhibit tumorigenesis through CCL, TPL, Nav and GDC0449 [122–125].

## Promoting tumorigenesis

As the bridge between growth factors and their corresponding receptors, TGFβ plays an important role in signaling pathways, e.g., the FOXO1/TGFβ1 signaling loop, IL1b/TGFβ-mediated crosstalk, the TGFβ1/SMAD3 signaling pathway, the TGF-β1/PI3K/AKT/mTOR pathway, and TGFβ accumulation [126–130]. In resistance to oxaliplatin, TGFβ signal transduction, an IL-1β antibody and a TGFBR1 inhibitor can drive TAK-mediated activation to decrease the JAK/STAT and PI3KCA/AKT pathways in CRC [127]. Furthermore, TGFβ1/SMAD3 activation, a fibrotic phenotype and nintedanib resistance were shown to be more strongly related to ADC than to SCC in lung carcinoma [128]. Moreover, in NSCLC, crosstalk activates the PI3K/AKT/mTOR pathway to induce the MDR by releasing TGF-β [129]. In another chemoresistance mechanism, the crosstalk between the tumor cells and CAFs promotes the activation and expression of FOXO1, which leads to TGFβ1 expression through autocrine/paracrine loops in ESCC. However, Yamei Chen et al. recently reported that ANXA1/FPR2 signaling could counteract the function of TGFβ in ESCC [123]. Recent research revealed TGFβ-myCAF signaling could be modulated by the EMILIN1 gene in IFNγ-iCAFs in breast cancer [125].

Many studies have demonstrated that IL-6-specific functions in CAFs involve the IL-6/CXCR7 axis and the IL-6/Jak1/STAT3 axis, which play roles in the immune response, EMT and cancer cell migration [131–134]. For DNA repair in ESCC, CXCR7 expression is increased by IL6 to protect cells from apoptosis via the activator of transcription 3/NF-κB pathway [131]. In addition, the monoclonal anti-IL-6R antibody tocilizumab increases apoptosis in cancer cells through the IL-6/Jak1/STAT3 axis, promoting tumorigenesis, invasion and antiapoptotic proteins expression in gastric cancer [132]. According to a recent study, in gastric cancer, in combination with M2 macrophages, eCAFs promote tumor invasion and decrease survival through the expression of periostin [122]. Because of anti-PD-L1 resistance in hepatocellular carcinoma, CAFs expressing high levels of IL-6 support the disruption of tumor-infiltrating T-cell function, resulting in the generation of immunosuppressive cells in the TME [133].

5-Fluorouracil and paclitaxel resistance can be induced by Snail-overexpressing fibroblasts, which secrete CCL1 via the TGFβ/NF-κB signaling pathway [135]. PAI-1, which is produced by inflammatory cells and CAFs, is involved in resistance in various tumor tissues. Research has demonstrated that AKT and ERK1/2 signaling is activated by PAI-1 and impedes the accumulation of ROS and caspase-3 activity during cisplatin resistance in ESCC [136] (Fig. 3). In addition, PAI-1 increases the expression of α-SMA, which is expressed in MFs, and reduces chemoresistance in lung cancer [137]. Recent studies have supported the resistance to antiandrogens in the EGFR/ERK pathway through miR-146a-5p and the NRG1/HER3 axis in prostate cancer; however, obstructing NRG1/HER3 and inhibiting migration and growth strengthens ADCC in NRG1-positive pancreatic tumors and CAFs [138, 139]. In addition, studies have shown that in pancreatic cancer, the SDF-1/CXCR4/SATB-1 axis induces tumor progression and GEM resistance by building a positive feedback loop [140].

For pancreatic cancer, the inhibitor of exosome release GW4869 suppresses epithelial cell proliferation and GEM resistance, as exosomes increase Snail expression, and Snail expression is correlated with the promotion of drug resistance in lung cancer [141, 142]. In addition, miR-106b, which targets to TP53INP1 and increases GEM resistance in pancreatic cancer, is released by exosomes from CAFs to tumor cells [143]. Another study reported that CAFs secrete exosomes decorated with miRNA-130a via PUM2 and inhibit apoptosis via the PKM2/BCL2 axis to promote cisplatin resistance in NSCLC [70, 144]. In CRC, exosomal miR-181d-5p, the direct target of NCALD, is affected by METTL3 via DGCR8 in CAFs, whereas the sensitivity of CRC to 5FU is affected by NCALD [145]. Recently, Arthur Dondi et al. revealed that lung cancer metastasis occurs through miR-1290/MT1G/AKT signaling in NFs [146].

### Inhibiting tumorigenesis

Recently, an increasing number of nanoparticle technologies that enhance therapeutic efficacy, inhibit signaling pathways, and reduce toxic side effects, have been developed for cancer treatment [147–150]. In gastric cancer, PSN38@TPL-Nsa nanoparticles have been shown to inhibit tumor progression, and improve antimetastatic efficacy because TPL is a suitable stromal reprogramming inducer [147]. In addition, nano-doxorubicin has been shown to improve drug efficacy and reduce cardiotoxicity in pancreatic cancer patients [150]. CCL plays crucial roles in chemoresistance, such as 5-fluorouracil resistance, paclitaxel resistance, and cisplatin resistance, in CRC, HNSCC, ovarian cancer and melanoma [151–153]. In addition, cisplatin resistance in ovarian cancer and HNSCC has been shown to occur via the regulation of the STAT3 and PI3K/Akt signaling pathways as well as sensitization effects of CAFs, respectively [151, 153] (Fig. 2). In addition, a recent study reported that EMT was increased by miR-146a-5p through the modulation castration resistance via the EGFR/ERK pathway [154] (Table 1).

**Table 1:** CAFs with drug resistance in different cancer types

Cancer Tissue	Targets	Therapy/Drug	Mechanism	Refs.
ESCC	PAI-1	Cisplatin	Extracellular PAI-1 activated the AKT and ERK1/2 signaling pathways and inhibited caspase-3 activity and reactive oxygen species accumulation.	[136]
ESCC	IL6-CXCR7 axis	cisplatin	IL6 derived from CAFs played the most important role in chemoresistance by upregulating CXCR7 expression via signal transducter and activator of transcription 3/NF-κB pathway.	[131]
ESCC	TGFβ1, FOXO1	cisplatin, taxol, irinotecan, 5-fluorouracil, carboplatin, docetaxel, pharmorubicin	The crosstalk of CAFs and ESCC cells enhanced the expression and activation of FOXO1, inducing TGFβ1 expression in an autocrine/paracrine signaling loop.	[126]
GC	IL-6	IL-6 inhibitors	IL-6 was prominently expressed in the stromal portion of GC tissues, and IL-6 upregulation in GC tissues was correlated with poor responsiveness to chemotherapy.	[132]
GC	Triptolide (TPL)	triptolide-naphthalene sulfonamide (TPL-nsa)	TPL significantly reduced CAFs activity and inhibited CAFs-induced chemotherapy resistance. PSN38@TPL-nsa treatment reduced the expression of collagen, FAP, and α-SMA in tumors.	[147]
CCA	IL-6, N-to E-cadherin switch	Resveratrol	While the CM from CAFs induced IL-6 mediated motility of CCA cells, the CM from CAFs pre-treated with Resveratrol halted cancer cell motility and reverted the N-to E-cadherin switch in migrating cells.	[134]
HCC	IL-6	anti-PD-L1	High IL-6 expression CAFs impaired tumor infiltrating T-cell function via upregulating inhibitory immune checkpoints. Using IL-6 blockade could reverse anti-PD-L1 resistance in HCC tumor model.	[133]
PDAC	SDF-1/CXCR4/SATB-1 axis	Gemcitabine	CAF-secreted SDF-1 upregulated the expression of SATB-1 in PDAC. SATB-1 knockdown inhibited proliferation, migration, and invasion in SW1990 and PANC-1 cells, whereas overexpression of SATB-1 in Capan-2 and BxPC-3 cells had the opposite effect.	[140]
PDAC	exosomes release	gemcitabine	CAFs exposed to gemcitabine significantly increase the release of EVs. These EVs increased chemoresistance-inducing factor, Snail, and promote proliferation and drug resistance.	[142]
PDAC	Exo-MiR-106b	Gemcitabine(GEM)	Suppressing miR-106b expression in CAFs-exosomes resulted in a decreased resistance to GEM.	[143]
PDAC	NRG1	anti-NRG1 antibody 7E3	The promising tumor growth inhibitory effect of the 7E3 promotes antibody dependent cellular cytotoxicity in NRG1-positive PC and CAFs and inhibits NRG1-associated signaling pathway induction, by blocking NRG1-mediated HER3 activation.	[139]
CRC	METTL3, miR-181d-5p axis.	5Fluorouracil	Exo-miR-181d-5p was identified as a miRNA associated with 5FU sensitivity. CAFsderived exosomes inhibited 5FU sensitivity in CRC cells through the METTL3/miR-181d-5p axis. miR-181d-5p directly targeted NCALD to inhibit the 5FU sensitivity of CRC cells.	[145]
CRC	TAK1, TGFBR1	oxaliplatin	TAK1 plus TGFBR1 inhibition blocks fibroblast activation and decreases the secretion of proinflammatory cytokines.	[127]
CRC	Snail-expression fibroblasts	5-fluorouracil, paclitaxel	CT26 co-cultured with 3T3-Snail resisted the impairment from 5-fluorouracil and paclitaxel. The subcutaneous transplanted tumor models included 3T3-Snail cells developed without restrictions even after treating with 5-fluorouracil or paclitaxel.	[135]
PCa	NRG1/HER3 axis	antiandrogen	Research identify NRG1 in CAF supernatant promoting resistance in tumor cells through activation of HER3. Pharmacological blockade of the NRG1/HER3 axis using clinical-grade blocking antibodies re-sensitizes tumors to hormone deprivation in vitro and in vivo.	[138]
PCa	exo-miR-146a-5p	Androgen deprivation therapy (ADT)	CAFs-derived exosomal miR-146a-5p confers metastasis in PCa cells under ADT through the EGFR/ERK pathway and it may present a new treatment for PCa.	[154]
BC	integrin αvβ3	protein (ProAgio)	CAFs and aECs in TNBC tumor express high levels of integrin αvβ3. The depletion of CAFs by ProAgio decreases intratumoral collagen and growth factors released from CAFs	[59]
LC	SNAI1 caf	GW4869	The level of SNAI1 in exosomes was crucial for inducing EMT in lung cancer cells. Treatment of CAFs with GW4869 inhibited their EMT-inducing effect on recipient epithelial cells.	[141]
LC	PAI-1	cisplatin	PAI-1 promotes pulmonary fibrosis through increasing MF characteristics, expressing α-SMA in fibroblasts. Besides, the effectiveness of cisplatin was increased by PAI-1 inhibitor.	[137]
LC	TGFβ1/SMAD2, SMAD3	nintedanib, antifibrotic drugs	The reduced fibrosis and nintedanib response of SCC-TAFs was associated with increased promoter methylation of the profibrotic TGFβ transcription factor SMAD3 compared with ADC-TAFs, which elicited a compensatory increase in TGFβ1/SMAD2 activation.	[128]
LC	miRNA-130a	cisplatin	Exo-miRNA-130a was transferred from CAFs to recipient NSCLC cells and knockdown of miRNA-130a reversed the effect of CAFs-derived exosomes in NSCLC cells.	[144]
HNSCC	caf	cisplatin	Increased expression of VEGFA, PGE2S, COX2, EGFR, and NANOG in cancer cells was characteristic for the increase of resistance. CCL2 expression was associated with sensitizing effect.	[151]
melanoma	Galectin-1, Osteopontin, CCL5, CCL9, BRAF	Ocoxin, Vemurafenib	Ocoxin interferes with the cell cycle, impairs the inherent and fibroblast-mediated melanoma cell migration, and reduces resistance to BRAF inhibition.	[152]

## Conclusion

To date, many studies have focused on components of the TME, such as CAFs, the ECM, and TAMs, rather than on tumor cells. Herein, we summarized the most recent studies regarding CAF-targeting therapy. Recently, we revealed cross-talk among miscellaneous cells and the spatial landscape in CRC, PDAC, BC. These findings have significant implications for comprehensively understanding the crosstalk between CAFs and cancer cells, the macroscopic spatial structure of CAFs and therapeutic targets in tumor tissue. Drug resistance, angiogenesis, metabolic reprogramming and tumor metastasis are four hallmarks of cancer. Findings thus far have identified promising therapeutic targets that should be developed in future studies. Taken together, more studies involving single-cell spatial transcriptomics sequencing should be conducted to obtain further knowledge of tumor progression, metastasis, drug resistance, angiogenesis, and metabolic reprogramming.

### Electronic supplementary material

Below is the link to the electronic supplementary material.

Supplementary Material 1

## Data Availability

No datasets were generated or analysed during the current study.

## Abbreviations

CAF: Cancer associated fibroblast
TILs: Tumor infiltrating lymphocytes
TIDCs: Tumor infiltrating dendritic cells
TAMs: Tumor associated macrophages
TANs: Tumor associated neutrophils
MDSCs: Myeloid derived suppressor cells
ST: Spatial transcriptomics
DSP: Digital spatial profiling
PDAC: Pancreatic ductal adenocarcinoma
GSEA: Gene set enrichment analysis
NL cell: Natural killer cell
MMP: Matrix metalloproteinase
GPNMB: Glycoprotein nonmetastatic B
TME: Tumor microenvironment
TGF: Transforming growth factor
IL: Interleukin
CCL: C-C motif chemokine ligand
PAI: Plasminogen activator inhibitor
NRG: Neuregulin
TPL: Triptolide
FOXO: Forkhead box protein
SMAD: The Caenorhabditis elegans SMA and MAD family
PI3K: Phosphoinositide 3-kinases
mTOR: the mammalian target of rapamycin
AKT: Protein kinase B
ADC: Lung adenocarcinoma
SCC: Lung squamous cell carcinoma
MDR: MultiDrug Resistance
ESCC: Esophageal squamous-cell carcinoma
CXCR7: C-X-C Motif Chemokine Receptor 7
JAK1: Janus kinase 1
STAT: Signal Transducer and Activator of Transcription
NF-κB: Nuclear Factor kappa B
ROS: Reactive oxygen species
EGFR: Epidermal growth factor receptor
ERK: Extracellular-regulated kinase
HER3: Human epidermal growth factor receptor 3
SDF-1: Stromal cell-derived factor-1
SATB-1: Special AT-rich binding protein
GEM: Gemcitabine
TP53INP1: Tumor protein 53-induced nuclear protein 1
PUM2: PUMILIO2
PKM2: The M2 isoform of pyruvate kinase
BCL2: B-cell lymphoma-2
NSCLC: Non-small-cell lung cancer
CRC: Colorectal cancer
NCALD: Neurocalcinδ
METTL3: N6‑methyladenosine (m^6^A) methyltransferase like 3
DGCR8: DiGeorge Syndrome Critical Region 8
5-FU: 5-Fluorouracil
PSN38@TPL-nsa: SN38 prodrug polymeric micelles@ triptolide-naphthalene sulfonamide
EMT: Epithelial–mesenchymal transition
ECM: Extracellular matrix
TNBC: Triple-negative subtype of breast cancer
FGF: Fibroblast growth factor
VEGF: Vascular endothelial growth factor
HGF: Hepatocyte growth factor
HUVEC: Human umbilical vein endothelial cells
MSC: Mesenchymal stem cells
MMP: Matrix metalloproteinases
EPA: Eicosapentaenoic acid
ATF4: Activating transcription factor 4
sEV: Small extracellular vesicles
ITGB4: Integrin beta 4
GFPT2: Glutamine-fructose-6-phosphate transaminase 2
MAPK: Mitogen-activated protein kinases
PFKFB3: 6-phosphofructo-2-kinase/fructose-2,6biphosphatase 3
CAV1: Caveolin1
MCT: Monocarboxylate transporter
LPA: Lysophosphatidic acid
HIF 1α: Hypoxia inducible factor 1α
LDHA: Lactate dehydrogenase A
ASPN: Asporin
LUAD: Lung adenocarcinoma
SDF-1: Stromal-derived factor-1
CRLM: Colorectal liver metastases
ACLY: ATP-citrate lyase
MTOC: Microtubule-organizing centers
ADAM: A disintegrin and metalloproteinase
TRAIL: TNF-related apoptosis-inducing ligand
BMP: Bone morphogenetic proteins
ITGBL 1: Integrin beta- like 1
JPJDR: Jianpi Jiedu Recipe
TNFAIP3: TNF alpha-induced protein 3
ZEB1: Zinc finger enhancer-binding protein 1
HOTAIR: HOX transcript antisense RNA
HMGB1: High mobility group box 1
