# Supplementary material for: Advances in targeting cancer-associated fibroblasts through single-cell spatial transcriptomic sequencing
Source: Biomark Res. 2024 Jul 29;12:73. doi: 10.1186/s40364-024-00622-9 (PMC11287900; doi:10.1186/s40364-024-00622-9)
Supplement: Supplementary file 1 — Supplementary Material 1 [file 40364_2024_622_MOESM1_ESM.pdf]

SPRINGER NATURE

Author Services

Editing Certificate

This document certifies that the manuscript

Advance in targeting cancer associated fibroblast through single-cell spatial transcriptomic sequencing

prepared by the authors

Pin Lyu, Xiaoming Gu, Fuqi Wang, Quanbo Zhou, Haifeng Sun, Shuaixi Yang, Weitang Yuan

was edited for proper English language, grammar, punctuation, spelling, and overall style by one or more of the highly qualified native English speaking editors at SNAS.

This certificate was issued on **July 1, 2024** and may be verified on the SNAS website using the verification code **2380-2B3D-95AB-18C3-708P**.

Neither the research content nor the authors' intentions were altered in any way during the editing process. Documents receiving this certification should be English-ready for publication; however, the author has the ability to accept or reject our suggestions and changes. To verify the final SNAS edited version, please visit our verification page at secure.authorservices.springernature.com/certificate/verify. If you have any questions or concerns about this edited document, please contact SNAS at support@as.springernature.com.

SNAS provides a range of editing, translation, and manuscript services for researchers and publishers around the world. For more information about our company, services, and partner discounts, please visit authorservices.springernature.com.
